# Sub-Pixel Extraction of Laser Stripe Center Using an Improved Gray-Gravity Method †

**DOI:** 10.3390/s17040814

**Published:** 2017-04-10

**Authors:** Yuehua Li, Jingbo Zhou, Fengshan Huang, Lijian Liu

**Affiliations:** School of Mechanical Engineering, Hebei University of Science and Technology, Shijiazhuang 050018, China; yuehua.hrbin@163.com (Y.L.); hfs_high@126.com (F.H.); liulijian2002@126.com (L.L.)

**Keywords:** line structured light sensor, improved gray-gravity method, adaptive sampling regions, uncertainty analysis, sub-pixel center extraction

## Abstract

Laser stripe center extraction is a key step for the profile measurement of line structured light sensors (LSLS). To accurately obtain the center coordinates at sub-pixel level, an improved gray-gravity method (IGGM) was proposed. Firstly, the center points of the stripe were computed using the gray-gravity method (GGM) for all columns of the image. By fitting these points using the moving least squares algorithm, the tangential vector, the normal vector and the radius of curvature can be robustly obtained. One rectangular region could be defined around each of the center points. Its two sides that are parallel to the tangential vector could alter their lengths according to the radius of the curvature. After that, the coordinate for each center point was recalculated within the rectangular region and in the direction of the normal vector. The center uncertainty was also analyzed based on the Monte Carlo method. The obtained experimental results indicate that the IGGM is suitable for both the smooth stripes and the ones with sharp corners. The high accuracy center points can be obtained at a relatively low computation cost. The measured results of the stairs and the screw surface further demonstrate the effectiveness of the method.

## 1. Introduction

As a non-contact measuring apparatus, the line structured light sensor (LSLS) can effectively obtain a huge amount of surface data from objects with the advantages of simple construction, low cost and moderate precision. It has been widely used for profile measurement [1], reverse engineering [2], welding inspection [3], visual tracking [4], etc. The principle of the LSLS is to project a linear laser stripe onto the cross-section profile to be measured, and then compute the profile’s world coordinates according to the stripe image that is captured by the camera and the system parameters from the calibration process [5]. The laser stripe usually has a width of several to tens of pixels and the center extraction is a critical step for the measurement process [6].

Many researchers have studied the center extraction methods aiming at better precision, higher efficiency, more robustness, and lower noise. According to the minimum coordinate value, these algorithms can be divided into two categories: pixel level center extraction and sub-pixel center extraction. The simplest pixel level center extraction method is the extreme value method. It is achieved just by choosing the pixel with the maximum gray value as the center of the specific transverse profile. Its disadvantages are also obvious: low precision and sensitive to noise [7]. The direction template [8] and the improved direction template methods [9] can also obtain the stripe center at the pixel level. The influence of noise on the center line extraction can be suppressed effectively, but these methods require a large amount of calculation due to the cross-correlation processes.

With the demand for higher measurement precision, the sub-pixel center extraction methods are receiving more and more attention. An important one is the gray-gravity method (GGM) which is simple and easy to use [10,11]. In contrast, due to its sensitivity to noise, the measurement results of the smooth profiles are coarse. Usamentiaga et al. proposed a fast center extraction method which is based on the GGM and the subsequent Split-and-Merge process [12]. It can be successfully applied for the 3D reconstruction of steel stripes and weld seam tracking. By setting a specific window, the preliminary center data can be smoother than that of the traditional GGM. However, the center extraction is achieved in the column direction, not in the normal direction of the stripe. This would introduce significant extraction error, especially for the steep stripe with uneven intensity distributions. Moreover, by adopting a fixed window width, the extracted points would have a large deviation at the sharp corner region. The FIR (Finite Impulse Response) filter approach also simply treats the direction of stripe’s cross section as the direction of the column/row [13]. Therefore, it is suitable for the smooth stripes with the normal vectors generally in the column/row direction. The curve fitting method that is based on the Laplacian of the Gaussian operator can be adopted to detect the center of the laser stripe [14]. To use this method, the distribution of the gray value in the cross-section direction is assumed to be symmetrical, but very often it is not.

The Steger method [15], which was originally designed to analyze the aerial and the medical images, is one of the commonly used methods for the center extraction of laser stripes thanks to its high robustness and accuracy [16]. During the center extraction process, all the image pixels need to be convolved with the Gaussian kernel five times, and the eigenvalues and the eigenvectors of each Heissen-matrix need to be solved to obtain the sub-pixel center [17]. This involves an enormous amount of computation and thus needs a long computation time. To achieve the real-time processing of the light stripe, Xu et al. used the Steger method to detect the first feature point of the stripe and then searched the left points by utilizing the local orientations of the light stripe [18]. Beside this, the GPU (Graphics Processing Unit) [19] or FPGA (Field Programmable Gate Array) [20] can also be applied for the center detection using the Steger method to further reduce the computation time. However, the center extraction result of the Steger method highly relies on the parameters of the extraction algorithm such as the standard deviation of the Gaussian kernel and the threshold of the eigenvalues [21]. With a fixed set of parameters, the Steger method is hardly adapted to the stripes with different widths and noise amplitudes. The center points from the Steger method may also be fitted using straight lines [22]. The linear fitting is only appropriate for inspecting planar surfaces.

There are also many other methods for the center extraction of the laser stripes. Cai et al. used principle component analysis to estimate the normal direction and then the sub-pixel center points were calculated by second order Taylor expansion [23]. This method is also based on the Gaussian convolution process and the computational expense is high. Sun et al. proposed a robust center extraction method which is based on the grey level moment and the smoothing spline algorithm [24]. This analysis is on the basis that the light intensity has a uniform distribution. While, the intensities of the cross section for most laser stripes follow the Gaussian distribution [17]. The cross-correlation based curve fitting method [25] and the multi-scale analysis method [26] both can robustly obtain the center coordinates of the stripe. The computation time is long due to the complex processes.

From the above analysis, it can be seen that the GGM and other similar methods are of high computation efficiency, and the convolution based methods can usually leads to better accuracy. Focusing on the sub-pixel precision, an improved gray-gravity method (IGGM) is proposed with a competitive computational efficiency. The center points of the laser stripe can be first computed using the GGM and then each point can be modified within an adaptive rectangular region. The sub-pixel coordinates with high accuracy can be successfully achieved.

## 2. Methods for Center Extraction 

### 2.1. Traditional Gray-Gravity Method (GGM)

Assume **I**_M×N_ is the image that contains the perturbed laser stripe. It is captured by the camera with the pixels of M rows and N columns. The gray value for the pixel at the *i*th row and *j*th column is denoted by I(*i*,*j*) and the maximum gray value of the image is I_max_. Thus, the normalized gray value for I(*i*,*j*) can be calculated as: I*_n_*(*i*,*j*) = I(*i*,*j*)/I_max_. The sequence number for the center point at the *j*th column is denoted as *p* with a center coordinate of C*_p_*(*x_p_*,*y_p_*). Using the GGM, the center coordinates can be computed as:

(1) $$C_{p}\left(x_{p},y_{p}\right)=\left(j,\sum_{j=1}^{M}I_{n}\left(i,j\right)\times i/\sum_{j=1}^{M}I_{n}\left(i,j\right)\right)$$

To successfully obtain the center coordinates using the GGM, the relationship between the camera and the linear laser projector should be carefully adjusted to guarantee that each column of the image only intersects with the stripe once at most. Some columns may not intersect with the stripe, so *p* is no larger than *N*. It can be seen from Equation (1) that the GGM is simple and therefore fast. Its disadvantages are also obvious: the center extraction is not in the transverse direction of the stripe and it is sensitive to noise.

### 2.2. Improved Gray-Gravity Method (IGGM)

**Figure 1:**
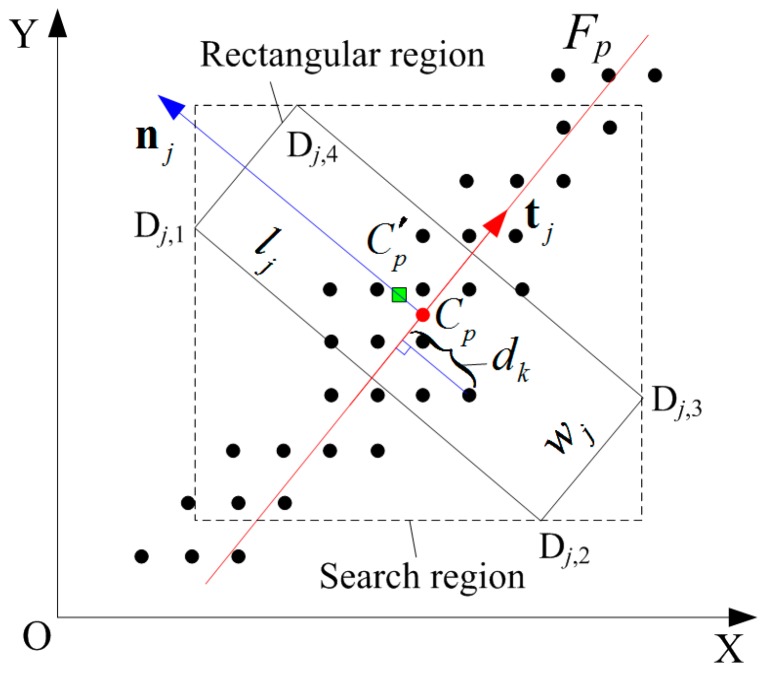
Principle for center extraction using the improved gray-gravity method (IGGM).

The computation principle of the IGGM is shown in Figure 1. The dots are the pixels of the image with I*_n_* larger than the threshold value T_0_. C*_p_* is the center coordinate for the *j*th column computed using the GGM; **n***_j_* and **t***_j_* are the normal and the tangential vectors at C*_p_*, respectively. *F_p_* denotes the line function that crosses C*_p_* and coincides with **t***_j_* expressed by: *Ax + By + C* = 0. The coordinate of the new center point C*_p_*^’^ computed using the IGGM is achieved in the rectangular region. The width of the rectangular region, *w_j_*, adapts to the radius of curvature at C*_p_*.

Sometimes, the stripe that is captured by camera is discontinuous. The center coordinates obtained by the GGM need to be divided into several sections based on the discontinuity points. In each continuous section, **n***_j_*, **t***_j_*, and the radius of curvature *ρ_j_* for C*_p_* are calculated using the moving least square method [27]. Assuming the fitting Equation is:

(2) $$f(x)=\sum_{i=1}^{m}\alpha_{i}p_{i}(x)=p^{T}(x)\alpha$$

where ***p***(*x*) = [*p*_1_(*x*), *p*_2_(*x*),…, *p_m_*(*x*)]*^T^* is a *m*th-degree basis function, and ***α*** = [*α*_1_, *α*_2_,…, *α_m_*]*^T^* is the vector of the undetermined coefficients. The weighted fitting error can be computed as:

(3) $$J(\alpha )={\sum_{j=1}^{n}\omega (r_{j})\left[p^{T}(x)\alpha -y_{j}\right]}^{2}$$

In function (3), *n* is the number of center points in the selected area named **C***_j_*; *r_j_* is the normalized distance between **C***_j_* and **C***_p_*, and can be computed as:

(4) $$r_{j}=\left\|C_{j}-C_{p}\right\|\cdot R_{s}^{-1}$$

where *R_s_* is the supporting radius of the fitting domain, and ω(*r_j_*) is a spline weighted function with fourth-degree, computed as:

(5) $$\omega (r_{j})=\{\begin{array}{ll} 1-6r_{j}^{2}+8r_{j}^{3}-3r_{j}^{4} & r_{j}\leq 1\\ 0 & r_{j}>1 \end{array}$$

The undetermined coefficients, ***α,*** can be obtained by solving the linear Equation of Ə*J*(***α***)/Əα*_l_* = 0, where *l* = 0,1,…,*m*. Using the fitting Equation (2), **n***_j_*, **t***_j_* and *ρ_j_* can be easily computed. The minimum radius of curvature is denoted as *ρ*_min_ and the maximum radius of curvature is *ρ*_max_.

The complexity of the laser stripes relies on the surface being measured. The stripe may contain arc segments, sharp corners and straight-line segments. For the straight-line portion, the value of its radius of curvature is infinite, while for the sharp corner, the radius of curvature is very small. If the width of the rectangular region, *w_j_*, linearly depends on *ρ_j_*, it could be extremely large. To limit *w_j_* in a reasonable range, a nominal radius of curvature is defined as:

(6) $${\bar{\rho }}_{\min}=\{\begin{array}{ll} \rho_{\min} & \rho_{\min}>1\\ 1 & \rho_{\min}\leq 1 \end{array}$$

(7) $${\bar{\rho }}_{\max}=s\cdot {\bar{\rho }}_{\min}$$

(8) $${\bar{\rho }}_{j}=\{\begin{array}{l} \begin{array}{ll} {\bar{\rho }}_{\min} & \rho_{j}\leq {\bar{\rho }}_{\min} \end{array}\\ \begin{array}{ll} \rho_{j} & {\bar{\rho }}_{\min}<\rho_{j}\leq {\bar{\rho }}_{\max} \end{array}\\ \begin{array}{ll} {\bar{\rho }}_{\max} & \rho_{j}>{\bar{\rho }}_{\max} \end{array} \end{array}$$

Furthermore, *w_j_* can be computed by

(9) $$w_{j}=\frac{{\bar{\rho }}_{j}-{\bar{\rho }}_{\min}}{{\bar{\rho }}_{\max}-{\bar{\rho }}_{\min}}\cdot w_{\max}+\frac{{\bar{\rho }}_{j}-{\bar{\rho }}_{\max}}{{\bar{\rho }}_{\min}-{\bar{\rho }}_{\max}}\cdot w_{\min}$$

where *s* is a scale factor; *w_max_* and *w_min_* are the upper and lower limits of *w_j_*, respectively. The length of the rectangular region, *l_j_*, is manually set according to the different surface reflective characteristics of the objects. To enhance the computation efficiency for C*_p_*^’^, a searching region can be defined based on the four corner coordinates of the rectangular region, named **D***_j_*_,1_(*x_j_*_,1_,*y_j_*_,1_), **D***_j_*_,2_(*x_j_*_,2_,*y_j_*_,2_), **D***_j_*_,3_(*x_j_*_,3_,*y_j_*_,3_) and **D***_j_*_,4_(*x_j_*_,4_,*y_j_*_,4_), as shown by Figure 1. However, only the pixels that are within the rectangular region contribute to the computation of the new center and the selection criteria for **I***_n_*(*i*,*j*) is:

(10) $$\{\begin{array}{c} \left(\left|D_{j,2}I_{n}\right|\times \left|D_{j,2}D_{j,1}\right|\right)\cdot \left(\left|D_{j,3}I_{n}\right|\times \left|D_{j,3}D_{j,4}\right|\right)\leq 0\\ \left(\left|D_{j,1}I_{n}\right|\times \left|D_{j,1}D_{j,4}\right|\right)\cdot \left(\left|D_{j,2}I_{n}\right|\times \left|D_{j,2}D_{j,3}\right|\right)\leq 0 \end{array}$$

When **I***_n_*(*i*,*j*) meets the selection criteria, its distance to *F_p_* can be calculated as:

(11) $$d_{k}=sign\cdot \frac{\left|A\cdot j+B\cdot i+C\right|}{\sqrt{A^{2}+B^{2}}}$$

If **I***_n_* is above *F_p_*, the sign is positive, otherwise it is negative. Then, the modified distance in the normal direction can be achieved by:

(12) $$d_{p}=\sum_{k=1}^{K}d_{k}\cdot I_{n}(i,j)/\sum_{k=1}^{K}I_{n}(i,j)$$

The new center $C_{p}^{\prime }(x_{p}^{\prime },y_{p}^{\prime })$ can be computed as:

(13) $$\{\begin{array}{c} x_{p}^{\prime }=x_{p}-d_{p}\cdot \sin\theta \\ y_{p}^{\prime }=y_{p}+d_{p}\cdot \cos\theta \end{array}$$

where *θ* is the angle between **t***_j_* and the X axis.

### 2.3. Model of Uncertainty Analysis

Because the pixel coordinates of the center points are obtained one by one in the transverse direction of the stripe using the same computing strategy, the uncertainty analysis is carried out only in one transverse section using the Monte Carlo method which is a numerical method and can be achieved by adding uniform random noise to the input data [28]. Most of the time, the intensity of the laser stripes in the transverse direction follows the Gaussian distribution [17] and can be expressed by

(14) $$I_{k}=H\cdot \exp\left\{-{\left(d_{k}-d_{0}\right)}^{2}/\sigma^{2}\right\}+a_{0}\varepsilon_{k}$$

where *H* is the amplitude of the laser stripe; *d*_0_ determines the position of the center; *d_k_* is the pixel coordinate of the *k*th pixel in the transverse direction; *σ* is the standard deviation; *ε_k_* is the noise value generated by a uniform random generator; and *a*_0_ is the amplitude; on this basis, the cross profile can be generated with various noise amplitudes.

For each cross section with different noise amplitudes, its center coordinates can be obtained using different center extraction methods and the extraction uncertainty can be computed as [29]

(15) $${\bar{d}}_{p}=\frac{1}{M}\sum_{r=1}^{M}d_{p,r}$$

(16) $$u(d_{p})=\sqrt{\frac{1}{M-1}{\sum_{r=1}^{M}\left(d_{p,r}-{\bar{d}}_{p}\right)}^{2}}$$

where ${\bar{d}}_{p}$ is the average value of the multiple sampling; *d_p,r_* is the *r*th sampling value; *M* is the sampling number; and *u*(*d_p_*) is the uncertainty value of center extraction.

## 3. Experiment Results and Discussion

### 3.1. Uncertainty Analysis for Center Extraction

**Figure 2:**
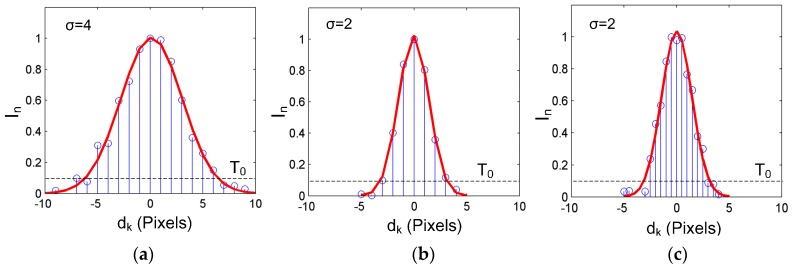
Normalized cross-section profiles at different simulation parameters: (**a**) *σ =* 4 and *s*_p_ = 1; (**b**) *σ =* 2 and *s*_p_ = 1; (**c**) *σ =* 2 and *s*_p_ = 0.2.

To analyze the uncertainty of the center extraction, the simulated transverse profiles of the laser stripe can be generated using Equation (14). For easy analysis, *d_0_* is assumed to be 0 and *H* = 1. Before the center calculation, the profile data should be normalized as I*_n_* = I*_k_*/max(**I**). Thus, only two parameters are left to determine the cross-section profile: *σ* and *a*_0_. The parameter *σ* determines the width of the laser stripe. With the decrease of *σ*, the cross section becomes smaller and smaller, as shown in Figure 2a,b. *a*_0_ is the amplitude of the uniform noise generator. The smaller *a*_0_ is, the better the intensity of the laser stripe conforms to the Gaussian distribution. In Figure 2, *a*_0_ was set as 0.05 and the threshold value T_0_ = 0.1. The normalized gray value that is smaller than T_0_ was set to be 0 for the subsequent computation.

Different from the traditional GGM which has a minimum point interval, *s*_p_, of 1 pixel across the stripe, the proposed IGGM computes the center coordinate by projecting the pixels of the nearby rectangular region onto the cross section. Thus, the number of points for the center computation increased significantly within the same width of the stripe and the *s*_p_ decreased, as shown in Figure 2c.

**Figure 3:**
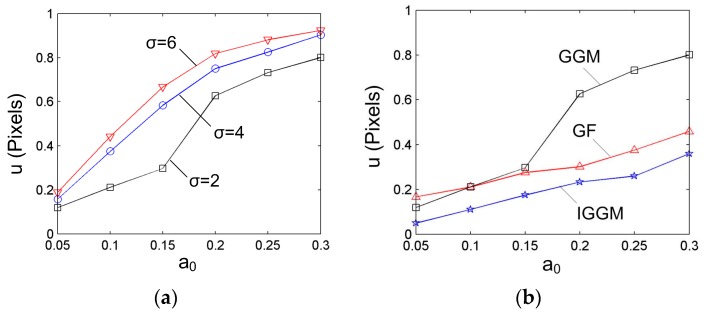
Uncertainty analysis for center computation: (**a**) Uncertainty values using the gray-gravity method (GGM) at different simulation parameters; (**b**) Comparison of the center uncertainty using different center extraction methods.

The uncertainty analysis was carried out by adopting different system parameters to the Gaussian distribution with a point step of 1, and the sampling number *M* = 4000; this means that for a specific cross section, its center was computed 4000 times at a given random generated noise amplitude. After that, the center coordinate was computed using the GGM, and the uncertainty value was computed using Equation (16). The results are shown in Figure 3a; they clearly indicate that the uncertainty of the center increases in accordance with the amplitude of the noise. For a given noise amplitude, the thinner the laser cross section, the smaller the center uncertainty will be.

Then, the width of the stripe was fixed (*σ =* 2), and the random generated noise with different amplitudes was added to the cross-section profile. Three different methods were used for the center computation. One is the GGM; its uncertainty values under different noise amplitudes have been obtained. The second method is the Gaussian fitting (GF), which is achieved by fitting the cross section using the Gaussian distribution Equation [30]. The third method is the proposed IGGM. The sampling number is *M* = 4000 for all of the cases. It should be noted that when the IGGM is adopted, the number of cross-section points is related with the width of the selected rectangular region. For most of the cases, the width of the adaptive region *w_j_* is within 2~8 pixels. Thus, the mean value of five pixels was chosen for the simulation which corresponds to a point interval of 0.2 pixels. The uncertainty values are shown in Figure 3b. From this figure, it can also be seen that the uncertainty values for the three methods increase with the noise amplitudes. Furthermore, the proposed IGGM can achieve smaller uncertainty values than the other two for all of the noise amplitudes. This validates the advantages of the proposed method.

### 3.2. Center Extraction of Laser Stripes

To extract the center using the IGGM, the traditional GGM should be firstly adopted to obtain the initial center point C*_p_* for each column. Then, the tangent vector, the normal vector and the radius of curvature for each point can be achieved via the moving least squares method. The base function of the moving least squares method is ***p***(*x*) = [*x*^3^, *x*^2^, *x*, 1]*^T^*; the supporting radius is *R_s_* = 5; and the scale factor is *s* = 5. Different from the GGM which only considered one cross-section profile for the center calculation, the IGGM would consider all the pixels within the rectangular region around *C_p_*.

**Figure 4:**
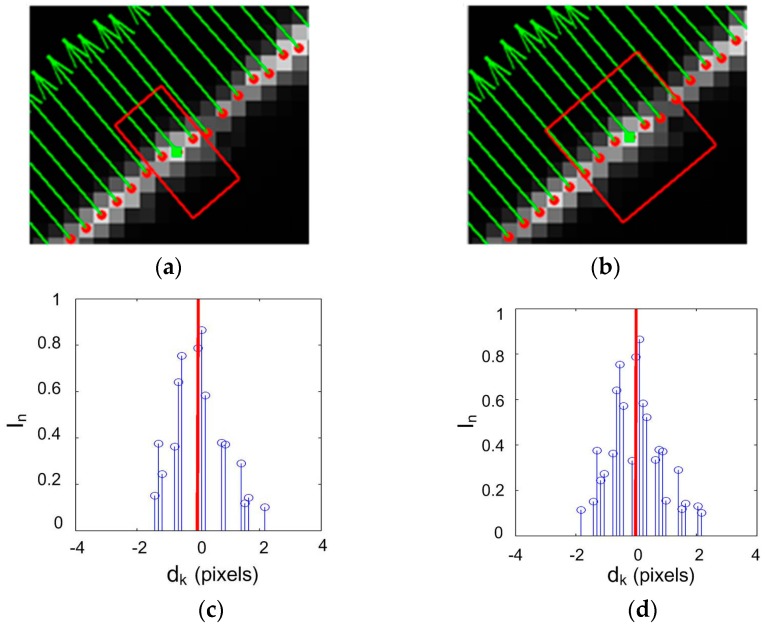
Center computation within a specific rectangular region: (**a**) rectangular region with *w_j_* = 4; (**b**) rectangular region with *w_j_* = 9; (**c**) pixels for **the** center computation of (**a**); (**d**) pixels for the center computation of (**b**).

To analyze the effluence of the width of the rectangular region, *w_j_*, on the center extraction, two rectangular regions were established for the same laser stripe and at the same initial center point, but with different width values, as shown in Figure 4a,b. For each pixel point within the rectangular region, its distance *d_k_* to the line Equation *F_p_* can be computed using Equation (11). Figure 4c,d denotes the gray pixels that were taken into account for the center computation. The offsets of the new centers in the normal direction are *d_p_* = −0.036 pixel, and *d_p_* = −0.022 pixel, respectively. From the uncertainty analysis, it can be seen that the uncertainty value of the center coordinates would become smaller with the increase of the width of the rectangular region.

**Figure 5:**
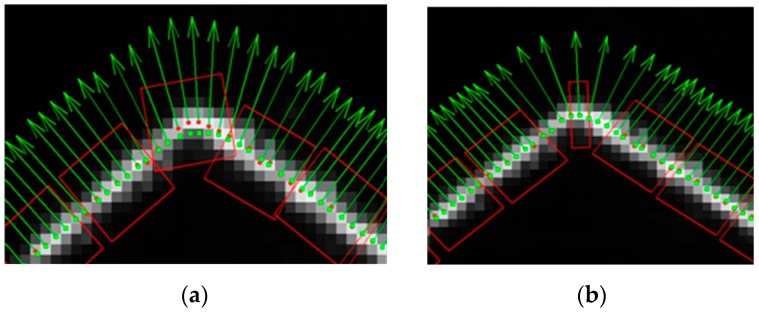
Center extraction at a sharp corner: (**a**) using rectagular regions with fixed width; (**b**) using rectagular regions with adaptive width.

For the actual center extraction process, the width of the rectangular region adapts to the radius of the curvature. To better show the advantages of the IGGM, a comparison experiment was carried out for a laser stripe with a sharp corner, as shown in Figure 5. In Figure 5a, the width of the rectangular region is fixed during the center extraction. In Figure 5b, the width decreased with the radius of the curvature accordingly. To clearly show the rectangular regions during the center extraction process, they were plotted every seven points. This figure shows that the recalculated centers are smoother than the GGM. When the width of the rectangular region is fixed, the extracted centers have a large deviation with the expected stripe center at the corner region. When using this proposed IGGM, fine extraction results can also be obtained at the corner region.

**Figure 6:**
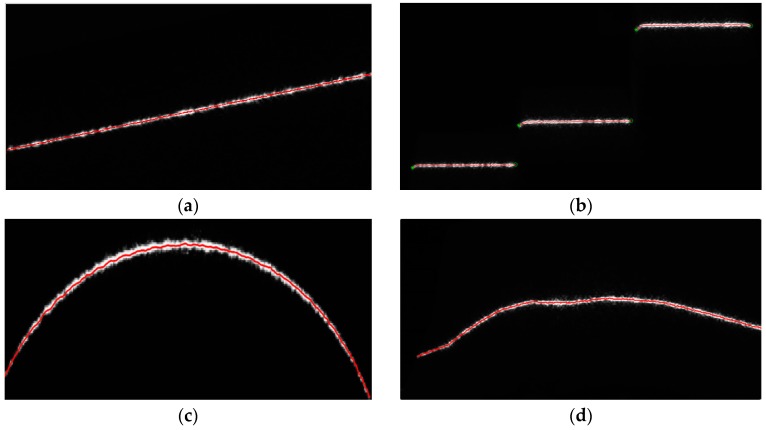
Center extraction results for different stripes: (**a**) the sloping straight line; (**b**) the steps; (**c**) the arc; (**d**) the random stripe.

The proposed IGGM can be applied for the center extraction of various laser stripes. Figure 6 lists four representative laser stripes together with the center extraction results. Though the complexity of the stripe increased accordingly, the obtained center curves of the stripes all conform well to the expected centers of the stripes. The quantitative analysis of the center extraction results can be found in the following sections.

### 3.3. Accuracy Analysis

**Figure 7:**
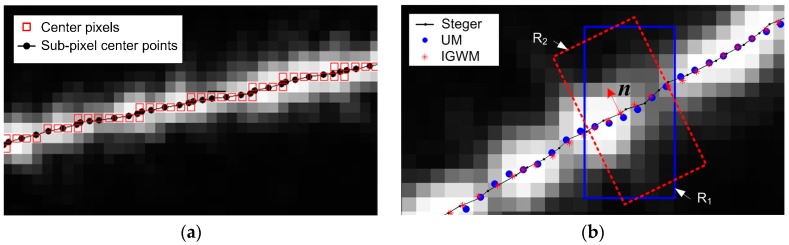
Center extraction results by different methods: (**a**) Steger method, (**b**) Comparision of the center extraction results.

The evaluation of center accuracy was conducted by comparing the center extraction methods with the classical Steger method which can reach sub-pixel accuracy when proper parameters are selected. Thus, it can be used as the reference for the accuracy evaluation. For this study, the sizes of the Guassian kernels are all 7 × 7, and their values are all generated out of the center extraction program to enhance the computation efficiency. Fine standard deviation of the Gaussian kernels and the threshold of the eigenvalues were found by many center extraction experiments to exclude the excess center points [21], and guarantee a fine center extraction result. The center pixels and the sub-pixel center points that were detected by the Steger method for Figure 6a, are shown in Figure 7a. It can be seen that although there may be multiple pixel responses to the stripe, their sub-pixel location conforms with the center perfectly.

Then, the center points of the stripe images in Figure 6 were computed by use of the Usamentiaga’s method (UM) and the proposed IGGM. It should be noted that, in order to analyze the sub-pixel center extraction accuracy, the Split-and-Merge approach was not employed. Figure 7b is an enlarged portion of the center extraction result of Figure 6d. In this figure, it can be seen that the center points obtained by the proposed IGGM are closer to the center curve computed by the Steger method, than that of the UM. This is because the center extraction of the UM computes the center coordinates in the column direction as illustrated by R_1_, not in the normal direction. The computation region of the IGGM denoted by R_2_, on the other hand, is along the normal direction which conforms with that of the Steger method.

To analyze the center extraction error quantitatively, the stripe center of the images in Figure 6 was computed by the Steger method, the UM and the IGGM, respectively. For the *p*th point that was obtained by the UM, its distance to the center curve of the Steger method is denoted as *e_p_*, and can be considered as the extraction error of this point. Then, the maximum deviation, the meam value and the root mean squares value of the UM can be easily computed. The center extraction error of the IGGM can be obtained using the same procedure as listed in Table 1. To better compare the accuracy of the center extraction, the relative error reduction (RER) from of the UM to the IGGM was also computed. 

It can be concluded from Table 1 that (1) the center extraction errors of the IGGM are all smaller than those of the UM; (2) the center extraction error is affected by the shape and the noise of the stripe. When the stripe lies in a horizontal direction and has low noise, as shown in Figure 6b, the center extraction error of the IGGM is a bit less than that of the UM. This is because the normal direction of the laser stripe is generally in the column direction which is the same as the UM. However, when the noise is increasing and the shape of the stripe is becoming complicated as shown in Figure 6c, the IGGM shows significant advantages over the UM. The three error evaluation parameters can all be reduced by more than 50%.

In addition, when the stripe has a sharp corner, the center points obtained by the UM would have a large deviation from the true stripe center, similar to that of Figure 5a. The deviation value grows with the width of the window for the center computation. Furthermore, this deviation error is hard to fix by the subsequent Split-and-Merge procedure. The IGGM, on the other hand, can alter the width of the window according to the radius of the curvature of the stripe. Thus, it can precisely obtain the center of the stripe both at the smooth region and the corner region.

### 3.4. Run Time

In this experiment, the computer for the image processing has a CPU of Intel i5-3470 3.2GHz and a RAM of 4GB. The software environment is Matlab R2012b. For ease of comparison studies, the sizes of the images of Figure 6 are all 500 × 250 pixels. For each image, the center extraction program was run 10 times, and the average times of different center extraction methods are illustrated in Table 2. From this table, it can be seen that the GGM is the most efficient and the Steger method always needs the longest computation time. This is because the GGM has the simplest computational strategies as shown by Equation (1), while the Steger method needs to convolve all the pixels of the image with the Gaussian kernels five times. The UM was realized by the center extraction and subsequent Split-and-Merge process. The window size for the center calculation is W = 4 and the Akiama splines are used for the fitting with a threshold of *ε* = 2. Unlike other methods, its center extraction time is related with the stripe images. The more complex the stripe is, the more interactive steps are needed to restrict the fitting error within the threshold, and the more computation time is needed.

The IGGM has a longer computation time than that of the UM. This is because the moving least squares method is applied to estimate the normal vector of each center point. Furthermore, the center extraction in the normal direction leads to better accuracy. Moreover, the computation time of the IGGM is less than 0.1 s which is only one thirtieth of the Steger method. Thus, it can be applied for the 2D profile measurement and also for the 3D shape measurements by combining it with other motional axes. A lower computation time can be expected by transferring the algorithm to another software environment.

## 4. 2D and 3D Measurement

### 4.1. 2D Profile Measurement

**Figure 8:**
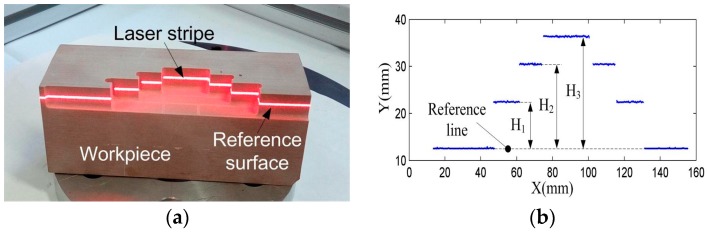
Measuring the stairs profile: (**a**) the workpiece with the stairs; (**b**) the measured results.

For accuracy evaluation, a workpiece with stairs was fabricated by a precision milling machine, as shown in Figure 8a. The LSLS was calibrated by using of the numerical calibration method [5]. It can effectively eliminate the effect of lens distortion on the measuring results. Besides this, the bottom face of the workpiece also needs to fit well with the supporting plane to make the stairs perpendicular to the laser plane. The measured stair profile is shown in Figure 8b. A reference line was firstly defined by linear fitting of the line segments from the reference surface. Then, the value of the stair heights, named H_1_, H_2_ and H_3_, are defined as the average distance between the stair points and the reference line. The profile was also inspected by CMM (Coordinate Measuring Machine). The height values were computed using the same procedure and were taken as the true value. The measuring results are shown in Table 3. It can be seen that the maximum deviation is 0.0352 mm and the maximum relative error is 0.1933%. This indicates the high accuracy of the LSLS.

### 4.2. 3D Measurement

**Figure 9:**
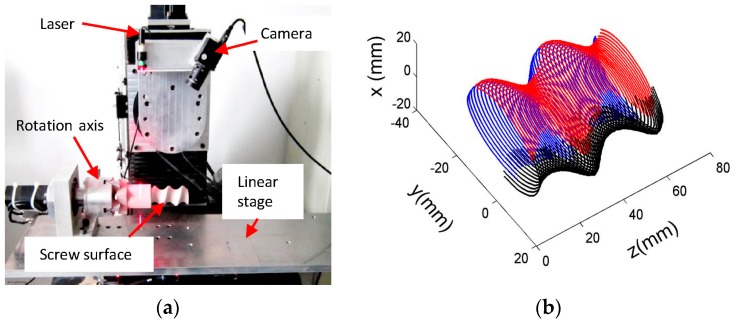
Measuring applications: (**a**) The system setup; (**b**) The measured screw surface.

The IGGM was also used to extract the center coordinates of the laser stripe during the measurement of a screw surface, as shown in Figure 9.

Figure 9a is the measurement system that consisted of a linear laser projector, a CCD camera, a rotation axis and a linear stage. The LSLS, which has a linear laser projector and a CCD camera, was well calibrated before assembling with the motional axes. For this study, two motional axes are used for the surface inspection. One is the rotation axis that is driven by a servo motor with a resolution of 0.0055°. Another is a linear stage from NSK Ltd. (Tokyo, Japan) with a resolution of 0.5 μm. Figure 9b is the measured result of the screw surface mounted on the rotation axis. The linear stage carries the screw surface linearly under the LSLS; the position of the stage and the cross profile of the rotor were recorded. Then, the rotation axis moves to the position of 120° and 240°, and the same procedure was carried out. The measurement results further prove that the proposed IGGM can also be used for the measurement of the complex 3D surfaces.

## 5. Conclusions

An IGGM was proposed which can precisely extract the laser stripe center at sub-pixel level. Using this method, each center coordinate was not obtained by considering only one transverse section of the laser stripe, but a rectangular region nearby. Based on the relationship established between the width of the rectangular region and the radius of curvature of the stripe, the width of the rectangular region would automatically change with the radius of curvature within a reasonable range. Thus, the precise sub-pixel center coordinates can be obtained from the perturbed stripe not only for the smooth region, but also for the region with sharp corners. From the Monte Carlo simulation, it can be seen that the uncertainty values for the center extraction are lower than the GGM and the GF under different noise amplitudes. The center coordinates of the sloping straight line, the steps, the arc, and the irregular laser stripe are all successfully extracted by use of the IGGM. Compared with the UM, the proposed IGGM can achieve higher center extraction accuracy. Its average run time is only one thirtieth of the Steger method. Thus, the IGGM is suitable for precision measurement of the workpieces. The measurement results of a stairs profile and a 3D screw surface further prove that the IGGM can be used for actual applications.

## Figures and Tables

**Table 1:** Center extraction error by the Usamentiaga’s method (UM) and IGGM (pixels).

No.	Maximum			Mean			Root Mean Square		
	UM	IGGM	RER	UM	IGGM	RER	UM	IGGM	RER
Figure 6a	0.908	0.662	27.1%	0.205	0.152	25.9%	0.266	0.192	27.8%
Figure 6b	0.752	0.723	3.9%	0.204	0.184	9.8%	0.264	0.241	8.7%
Figure 6c	2.805	1.325	52.8%	0.661	0.267	59.6%	0.799	0.345	56.8%
Figure 6d	1.196	0.960	19.7%	0.249	0.205	17.7%	0.328	0.268	18.3%

**Table 2:** Average run time for the center extraction (s).

No.	Steger	GGM	UM	IGGM
Figure 6a	2.806	0.006	0.015	0.091
Figure 6b	2.811	0.006	0.023	0.089
Figure 6c	2.802	0.005	0.020	0.095
Figure 6d	2.819	0.006	0.047	0.096

**Table 3:** Measured results of the stairs.

No.	CMM (mm)	LSLS (mm)	Error (mm)	Relative Error (%)
H_1_	10.0021	9.9851	−0.0170	−0.1700
H_2_	18.0027	18.0375	0.0348	0.1933
H_3_	24.0039	23.9687	−0.0352	−0.1466
